# Evolution of porous materials from ancient remedies to modern frameworks

**DOI:** 10.1038/s42004-021-00549-4

**Published:** 2021-08-05

**Authors:** Gregory S. Day, Hannah F. Drake, Hong-Cai Zhou, Matthew R. Ryder

**Affiliations:** 1 Neutron Scattering Division, Oak Ridge National Laboratory, Oak Ridge, TN USA; 2 Department of Chemistry, Texas A&M University, College Station, TX USA; 3 Department of Materials Science, Texas A&M University, College Station, TX USA

**Keywords:** History of chemistry, Metal-organic frameworks

## Abstract

Porous materials play a significant role in modern chemistry and materials science; despite recent scientific interest, they have a history dating back to antiquity. Here the authors provide a brief overview of the past that has contributed to their evolution.

Scientific interest in porous materials has witnessed exceptional growth over the past few decades with the development of modern frameworks. However, it is important to appreciate that porous materials have been around for longer than we might initially think.

Reading about porous materials used in ancient Egypt for medicinal purposes ignited our curiosity about the history of this field. A quest for primary sources then led us down somewhat of a rabbit hole, but an enjoyable one nonetheless. We, therefore, aim to provide a brief chronicle of the evolution of porous material discovery from ancient remedies to modern frameworks (Fig. 1).

**Fig. 1:**
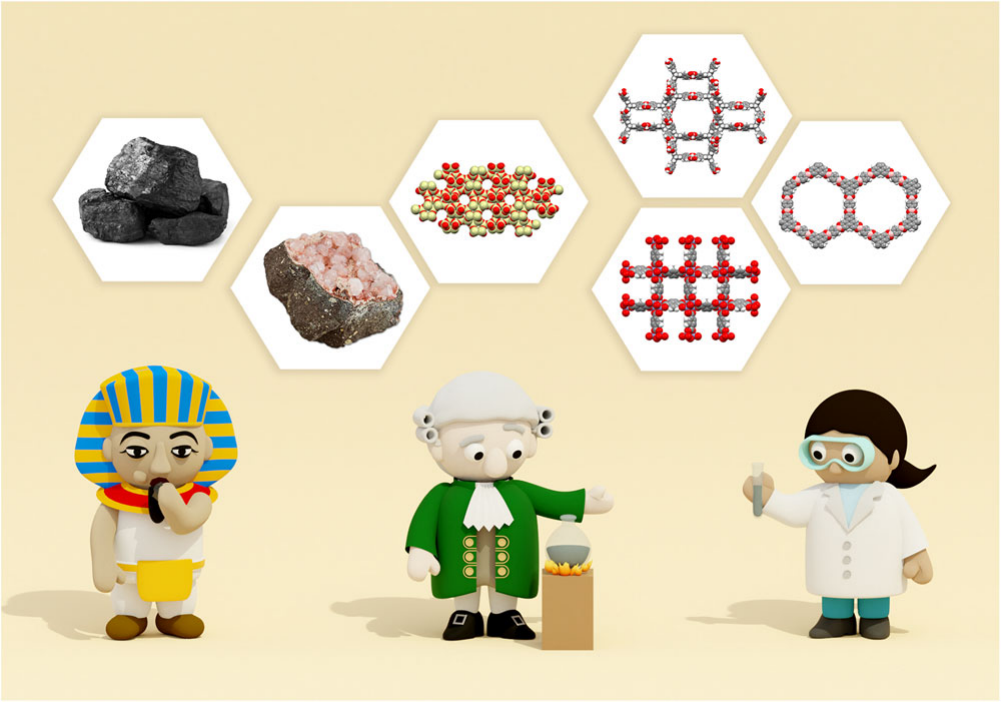
A brief illustration of the evolution of porous materials through the ages. Ancient civilizations throughout the world used porous materials for medicine (left), and in the 18^th^ century (middle), scientific study of these materials began. Modern scientific research has benefited and built upon this historical field and developed next-generation materials for applications such as carbon capture and clean energy (right). Note: Photographs of coal and natural zeolite used with the permission of Getty Images.

Scientific literature dating back to ancient Egyptian times is unsurprisingly scarce, as even the sturdiest papyri degrade over time. However, reports dating back to circa 1500 BC, in the Ebers papyrus, describe medical practices using porous charcoal for indigestion^1^. This describes the consumption of Egyptian ink, a mixture of charcoal suspended in gum Arabic slurry^2^.

The practical use of charcoal for its absorptive properties continued throughout antiquity and into the early modern era to treat gastrointestinal diseases. Pliny the Elder quoted the older Roman Scholar, Varro, “*let the hearth be your medicine-box*” when discussing charcoal^3^. The purification of water with charcoal is also reported in ancient Hindu sources^4^. The British navy, during early exploration, also used to char the interior of wooden barrels to improve the shelf life of potable water. However, the charcoal stained the water, making it less desirable^5^. The consumption of charcoal in the animal kingdom has also been observed, with theories suggesting that Zanzibar red colobus monkeys use it for the adsorption of phenolic compounds^6^. There is even evidence of charcoal consumption in a nodosaurid ankylosaur dinosaur specimen of the genus Borealopelta from the early cretaceous period^7^, although unfortunately, we do not know for sure if the consumption was intentional or not. Charcoal is still currently used as a feed additive for livestock to improve growth and health^8^. Other porous materials like kaolinite, a clay mineral, have been used by humans throughout the world for antidiarrheal properties, including the commercial medication Kaopectate^9^. Even into the late 20^th^ century, raw kaolinite clays were sold in western African markets as oral antidiarrheal medicines^10^.

However, to start thinking of the scientific theories of absorption using porous materials, we must jump to 18^th^ century Europe where Carl Scheele, a Swedish pharmaceutical chemist, studied the adsorption of gas within charcoal^11^. Scheele observed that upon heating in a vessel attached to a rubbery bladder, charcoal expelled adsorbed gases. He noted that the expansion of the bladder was well beyond that typically observed for heating a sealed vessel.

Through the 18^th^ and 19^th^ centuries, there became a need for advanced filtration and purification systems. In particular, sugar from Caribbean plantations required excess refining^12^. Additionally, purification of the stomach through the adsorption of ingested poisons as anecdotes was widely reported. Some examples included the ingestion of arsenic trioxide by Michel Bertrand in 1811 and strychnine by Pierre-Fleurus Touéry around 1852, both followed by charcoal consumption^13^.

Meanwhile, in Tennessee, home of Oak Ridge National Laboratory (ORNL), the Lincoln County Process (as it would come to be known) used charcoal filtering to produce authentic Tennessee whiskey^14^. Carbon-based adsorptive materials gained popularity as the growing knowledge of germ theory in the late 19^th^ century made beverage purification an increasingly hot topic^15^. Charcoal eventually made its way into the gas masks of World War I due to its improved adsorption capacity over the traditional cotton or fiber adsorbents^16^.

The second half of the twentieth century saw a surge of interest in science and technology. While charcoal was not as widely regarded as some of the new materials coming into the picture, researchers continued to study the essential absorptive properties. Perhaps one of the most important was studying its chemical structure, performed via X-ray diffraction by Rosalind Franklin^17^. Best known for her contributions to DNA structure, Franklin was also played a significant role in the modern scientific understanding of porous carbons, having conducted several analyses on these materials during the 1940s^18^.

At the same time, as some of the early research into charcoal adsorption, another class of porous materials, known as zeolites (derived from Greek *“zeo”* (to boil) and “*lithos”* (stone)), were gaining popularity in the scientific community. Natural zeolites, aluminosilicate mineral derivatives with highly ordered pores, were discovered in 1756 by Axel Fredrick Cronstedt, a Swedish mineralogist, also credited with the discovery of elemental nickel^19^. During his analysis, Cronstedt observed that upon heating two samples, mixtures of stilbite and stellerite, one from a mine in Sweden and one from Iceland^20^, he observed steam production. This sign we now recognize as zeolitic pores desorbing water^19^.

Within a few decades of William Henry Bragg and William Lawrence Bragg discovering X-ray diffraction, there were already reports using this new structural determination technique to analyze zeolites^21^, clays^22^, and carbons. Some of the earliest structural studies of porous materials were conducted in 1930 by future two-time Nobel Prize winner (Chemistry and Peace) Linus Pauling, who studied sodalite^21^ and the clay mineral mica^23^.

The first synthetic zeolites, lévyne or levynite, were produced over a century after their first discovery, in 1862 by Henri Sainte-Claire-Deville^24^. However, there was still little interest in these niche materials until Richard Barrer established the field of modern synthetic zeolite research in the 1940s^25^. Following on from Barrer’s work, in 1948, Robert M. Milton began studying industrial zeolite synthesis under the Union Carbide company^26^. Milton produced zeolites from soluble silicon and aluminum precursors, characterized by the recent adaptation of powder X-ray diffraction techniques, allowing quick and easy screening of synthesized materials. In 1951, Milton started pushing for the study and use of zeolites as catalysts because of their strong adsorptive properties and atomically precise chemistry^26^. Their initial work showed zeolites could be highly beneficial as hydrocarbon cracking catalysts, and by 1959, Zeolite Y was being used as a hydrocarbon isomerization catalyst^26^. This development resulted in a general explosion of zeolite research, with many highly regarded scientists, such as Donald Breck, Jule Rabo, and Edith Flanigen, studying them for the commodity chemical industry. Zeolite processes developed in the past fifty years include methanol to olefins and acid-catalyzed aromatic alkylation^27^.

Silica aerogel formation reports predate the widespread adoption of synthetic zeolites, with Samuel Kistler allegedly developing the first aerogel as part of a bet made with Charles Learned in 1931^28,29^. Subsequently, more ordered mesoporous silicas were developed, namely the Mobile Composition of Matter (MCM) and Santa Barbara Amorphous (SBA) series of materials. Two notable examples, MCM-41^30^ and SBA-15^31^, were discovered in the 1990s and exceeded the pore size limits of zeolites (~2 nm) while taking advantage of the robust chemical benefits of silica.

Porous polymer networks (PPNs) also emerged around the late 1940s, with structures based on non-intrinsically porous polymeric systems of polystyrenes and sulfonated polystyrenes but were not heavily studied until the 21^st^ century. PPNs take advantage of rigid organic functional group moieties of well-known geometries. Neil McKeown, an early PPN adsorption pioneer who first dubbed the term polymers of intrinsic microporosity (PIMs)^32^, generated materials with specific pore sizes and high gas adsorption capacities.

By the late 1980s, it was apparent that coordination complexes and coordination polymers could be highly crystalline. Much of the early work on 2D and 3D crystalline coordination polymers came from Richard Robson^33^. Later, Susumu Kitagawa advanced the field by designing porous hybrid inorganic-organic materials throughout the 1980s and 1990s^34^. The development of porous coordination materials then grew in popularity with the development of stable and permanently porous metal-organic frameworks (MOFs) by Omar Yaghi in the late 1990s^35^.

MOF materials are promising candidates for gas adsorption, especially for carbon dioxide, methane, and hydrogen uptake. Recently, Omar Farha developed a material that reached the U.S. Department of Energy’s target performance for volumetric and gravimetric methane storage^36^. The explosion of interest in MOFs has also produced new materials based on many of the same principles, such as highly ordered porosity controlled through the self-correction of non-covalent interactions. Porous coordination cages (PCCs), first discovered in 1990 by Makoto Fujita^37^, are molecular analogs and allow for easy processability through solution-based approaches. The concept of PCCs was later combined with PIMs to develop permanently porous organic cages^38^.

Early catalytic applications of MOFs have focused on the metal centers or nodes of the material. The reactions primarily utilize the metal cations of the framework as Lewis acid catalysts. However, there was an understanding that the constrained pore sizes and organic functional groups could be used for chiral selectivity for enantioselective catalysis^39^. Further advancement in MOF catalysis came about not through the study of metal-based catalytic properties but by developing post-synthetic modification (PSM) in the mid-2000s^40^. PSM allows for the accessible introduction of functionality that can be utilized to perform additional chemical reactivity. There has also been growing interest in using MOF scaffolds for templation. The development of MOF-derived carbons (MOFdCs) was achieved by exploiting the high porosity of the parent structure as a template for producing highly stable, well-defined porous carbon^41^.

Other modern porous materials include covalent organic frameworks (COFs)^42^ and hydrogen-bonded organic frameworks (HOFs). The former is an emerging class of non-metal-containing analogs of MOFs. They show promise for many of the same applications but with the advantage of a fully covalently bound 2D or 3D organic structure, often with high levels of crystallinity and selectivity. The latter are a promising class of molecular materials that form porous frameworks through non-covalent hydrogen-bonding interactions^43^. These interactions often reduce the rigidity of highly porous HOFs, but many are soluble in organic solvents and, therefore, more processable and more readily regenerated in solution.

In many ways, the porous materials used in the pre-modern era seem unsophisticated and straightforward compared to the current state-of-the-art materials made with molecular-scale precision. However, despite the increase in material complexity, we are still using many of these materials for similar reasons. New experimental and computational techniques have enabled recent advances, improving our understanding and control of materials at the molecular level. In recent years, new techniques such as neutron scattering, pair distribution function analysis, high-performance computing, and cryogenic electron microscopy (cryo-EM) have allowed for a better understanding of the structure and dynamics of materials at the molecular level. For example, cryo-EM allowed for the first direct visualization of gas molecules loaded into the pores of a MOF^44^. This proves that even in a field as old as porous materials, there are always new things to learn, and it is never too late to look back at some of the materials studied by previous generations with fresh eyes.
